# Impact of Intravitreal Ranibizumab on Retinopathy of Prematurity: A Retrospective Analysis

**DOI:** 10.7759/cureus.105794

**Published:** 2026-03-24

**Authors:** Fumihiko Nitta, Hiroshi Kunikata, Shimpei Watanabe, Toru Nakazawa

**Affiliations:** 1 Ophthalmology, Tohoku University, Sendai, JPN; 2 Perinatal Medicine, Tohoku University, Sendai, JPN

**Keywords:** extremely low birth weight, intravitreal ranibizumab, retinal detachment, retinal photocoagulation, retinopathy of prematurity

## Abstract

Purpose

The purpose of this study was to evaluate the impact of intravitreal ranibizumab (IVR) introduction on treatment patterns for retinopathy of prematurity (ROP) and the incidence of severe complications, including retinal detachment (RD), in a real-world setting.

Methods

We retrospectively analyzed 785 infants born at ≤1800 g or ≤34 weeks of gestation between January 2016 and December 2022 at two tertiary hospitals in Japan. IVR (0.2 mg/0.02 mL) was introduced in August 2020 at Tohoku University Hospital and in December 2019 at Miyagi Children's Hospital. Infants meeting Early Treatment for Retinopathy of Prematurity (ETROP) criteria received either retinal photocoagulation (RPC), IVR, or both, depending on the treatment era. Severe cases with RD (stage 4A or higher) were managed with scleral buckling or vitreous surgery as needed.

Results

Of the 472 infants treated prior to IVR introduction, 41.3% were extremely low birth weight (ELBW), comparable to 41.5% among the 291 infants treated after IVR introduction. The proportion undergoing RPC significantly decreased from 9.7% to 5.4% following IVR availability (p=0.03). Notably, the incidence of RD dropped from 2.1% to 0% after IVR introduction (p=0.01), despite no significant change in ELBW distribution.

Conclusion

The introduction of IVR has markedly shifted ROP treatment paradigms, reducing reliance on RPC and completely eliminating RD in our cohort. IVR offers a targeted, less destructive therapeutic alternative, potentially preserving retinal architecture and improving long-term visual outcomes. While these findings are encouraging, prospective studies are needed to further assess the safety and efficacy of IVR in this vulnerable population.

## Introduction

Retinopathy of prematurity (ROP) remains a leading cause of childhood blindness worldwide [1]. Recent advancements in anti-vascular endothelial growth factor (anti-VEGF) therapies have revolutionized their management, providing viable alternatives to conventional retinal photocoagulation (RPC) [2-5]. However, to the best of our knowledge, there are no published studies on PubMed that compare the outcomes before and after the introduction of intravitreal ranibizumab (IVR) for the treatment of ROP. This study aims to investigate, in actual clinical practice, the impact of IVR on the incidence of severe ROP cases with retinal detachment (RD; stage 4A or higher), as well as the evolution of RPC in the treatment of ROP.

## Materials and methods

This retrospective, multicenter cohort study included 785 infants with a birth weight ≤1800 g or a gestational age ≤34 weeks who were admitted to the neonatal intensive care units at Tohoku University Hospital and Miyagi Children's Hospital between January 2016 and December 2022. All eligible infants underwent routine ophthalmologic screening for ROP according to institutional protocols. Infants were included if they had sufficient ophthalmologic follow-up to determine treatment indication and clinical course. Infants with incomplete ophthalmologic records or insufficient follow-up data were excluded. ROP was classified according to the International Classification of Retinopathy of Prematurity, Third Edition (ICROP3) [6]. Treatment indications were determined based on the Early Treatment for Retinopathy of Prematurity (ETROP) criteria [7].

Ophthalmologic examinations were performed using binocular indirect ophthalmoscopy after pharmacologic pupil dilation. Final ROP staging and treatment decisions were determined by two experienced retinal specialists (FN and HK). The timing of initial and follow-up examinations was determined according to postmenstrual age and clinical condition, in accordance with established screening guidelines. Follow-up examinations were continued until complete vascularization, regression of ROP, or progression requiring treatment or surgical intervention; infants who required treatment were followed for at least one year.

IVR was introduced at Tohoku University Hospital in August 2020 and at Miyagi Children's Hospital in December 2019. Prior to the availability of IVR, RPC was performed as the standard treatment for all infants meeting ETROP criteria. Following the introduction of IVR, treatment options included IVR monotherapy, RPC, or combination therapy, depending on the severity and location of ROP, presence of plus disease, and systemic condition of the infant, as determined by the retinal specialists. In particular, IVR was mainly administered to infants presenting with plus disease. Intravitreal injections were performed under sterile conditions in the neonatal intensive care unit. Ranibizumab (0.2 mg/0.02 mL) was injected into the vitreous cavity via the pars plicata using a 30-gauge needle, with care taken to avoid lens injury. Post-treatment follow-up examinations were performed to monitor regression, recurrence, or progression of ROP. RPC was performed under appropriate anesthesia, targeting the avascular peripheral retina using laser photocoagulation. In cases progressing to stage 4A or higher, surgical intervention, including scleral buckling or vitreous surgery, was performed as clinically indicated. The surgical approach was selected based on the extent and configuration of RD. Clinical data were retrospectively collected from medical records and included birth weight, gestational age, sex, treatment modality, and surgical interventions. The primary focus of this study was the occurrence of RD; therefore, other ROP-related complications were not systematically evaluated, while treatment-related parameters, including RPC use and burn count, were assessed.

Statistical analyses were performed using Excel Statistics version 4.05 (Microsoft, Redmond, Washington). Total RPC burn count per eye was analyzed using the Mann-Whitney U test, and categorical variables were analyzed using Fisher's exact test (two-sided). A p-value of <0.05 was considered statistically significant.

## Results

**Figure 1:**
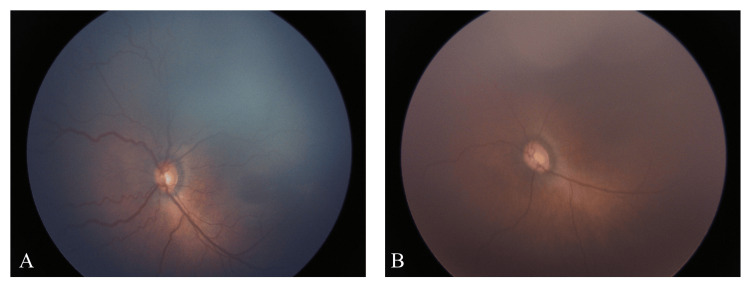
Representative eye with ROP before and after IVR A monochorionic diamniotic twin was born at 25 weeks and one day of gestation (birth weight: 678 g). At 34 weeks of corrected gestational age, IVR was performed for stage 2+ ROP in zone 2, in accordance with the International Classification of ROP (Figure 1A). Six days after treatment, resolution of plus disease was observed (Figure 1B). IVR - intravitreal ranibizumab; ROP - retinopathy of prematurity

As shown in Table 1, among the 472 infants treated before the introduction of IVR, 195 (41.3%) were extremely low birth weight (ELBW) infants. Similarly, 130 (41.5%) of the 291 infants treated after the introduction of IVR were ELBW, indicating no significant difference between the groups. The number of cases undergoing RPC decreased significantly following the introduction of IVR, from 46 infants (9.7%) to 17 infants (5.4%) (p=0.03). In addition, the total RPC burn count per eye decreased significantly after the introduction of IVR, from 2031.1 ± 1369.4 to 1369.4 ± 1363.6 (p=0.03). Notably, the number of cases resulting in RD (stage 4A or higher) after IVR and/or RPC decreased from 10 infants (2.1%) before IVR to 0 infants (0%) after its introduction (p=0.01). A representative eye with ROP successfully treated with IVR is shown in Figure 1.

**Table 1:** Characteristics of ROP Before and After the Introduction of IVR Total RPC burn count per eye was analyzed using the Mann–Whitney U test, and categorical variables were analyzed using Fisher's exact test (two-sided). IVR - intravitreal ranibizumab; ELBW - extremely low birth weight; RPC - retinal photocoagulation; SD - standard deviation; RD - retinal detachment

	Before the introduction of IVR	After the introduction of IVR	p-value
Total number of cases	472	313	
Number of ELBW infants (%)	195 (41.3%)	130 (41.5%)	1.00
Treatments			
Number (%) of cases that received IVR	0 (0%)	11 (3.5%)	-
Number (%) of cases that received RPC	46 (9.7%)	17 (5.4%)	0.03
Total RPC burn count per eye (mean ± SD)	2031.1±1369.4	1369.4±1363.6	0.03
Number of cases resulting in RD: Stage 4A or higher (%)	10 (2.1%)	0 (0%)	0.01

## Discussion

The IVR has significantly transformed the management of ROP, representing a paradigm shift from conventional RPC toward targeted pharmacologic therapy. In the present study, the introduction of IVR was associated with a substantial reduction in both the frequency of RPC and the number of laser shots required. Importantly, this change occurred despite no significant difference in the proportion of ELBW infants between the pre-IVR and post-IVR periods, suggesting that the observed shift in treatment patterns was primarily attributable to the availability of IVR rather than differences in baseline disease severity.

RPC has long been established as an effective treatment for ROP by ablating the avascular peripheral retina and reducing angiogenic drive. However, this therapeutic effect is achieved at the cost of permanent retinal destruction and scarring, which may adversely affect retinal structure and function [8,9]. Laser-induced retinal damage can compromise peripheral visual field development and interfere with normal retinal maturation. Furthermore, previous studies have demonstrated an association between extensive laser treatment and the development of high myopia [10-12], likely due to altered ocular growth and disruption of normal retinal signaling pathways. Therefore, reducing the extent of laser treatment may contribute to improved long-term visual and refractive outcomes.

In contrast, IVR selectively inhibits VEGF, which plays a central role in the pathological neovascularization characteristic of ROP. By targeting the underlying molecular mechanism of disease, IVR suppresses abnormal angiogenesis while preserving retinal tissue integrity. This tissue-sparing effect allows continued physiologic vascular development and minimizes irreversible retinal damage. The reduction in both the frequency and extent of RPC observed in this study suggests that IVR may preserve retinal structure and function more effectively than conventional laser therapy, thereby supporting improved visual development.

Another important finding of this study was the absence of RD following the introduction of IVR. RD represents one of the most severe complications of ROP and is generally associated with poor visual prognosis, particularly in advanced stages, even with surgical intervention [13,14]. Preventing progression to advanced stages of ROP is therefore critical for preserving visual function. Previous reports have demonstrated that anti-VEGF therapy effectively reduces disease progression and lowers the risk of RD [15]. The present findings are consistent with these observations and suggest that IVR contributes to improved structural outcomes in infants with treatment-requiring ROP. In addition to its anatomical benefits, IVR offers practical clinical advantages. Compared with laser photocoagulation, intravitreal injection is less invasive, requires less procedural time, and may impose less systemic stress on medically fragile premature infants. This may be particularly beneficial in infants with systemic instability, in whom prolonged procedures may pose additional risks.

Despite these promising findings, several limitations should be acknowledged. First, this study was retrospective in design, which may introduce selection bias and limit causal inference. In addition, stage-matched comparisons between the pre-IVR and post-IVR periods were not performed, and retinal examinations were conducted by two experienced ophthalmologists, which may have introduced some variability in ROP staging; however, this variability was likely minimized. Second, long-term functional outcomes, including visual acuity, refractive status, and visual field development, were not evaluated. Furthermore, although IVR is administered locally, systemic absorption and potential effects on circulating VEGF levels remain a concern in premature infants, as VEGF plays an important role in organ development [16]. Therefore, continued long-term monitoring is essential to fully evaluate the systemic safety profile of IVR. Prospective, longitudinal studies are needed to assess long-term visual, anatomical, and systemic outcomes associated with IVR therapy. These findings support the growing role of anti-VEGF therapy as a first-line treatment option for selected cases of ROP. As clinical experience with IVR continues to expand, this treatment modality may further improve both structural and functional outcomes in infants with ROP.

## Conclusions

The introduction of IVR has significantly altered the treatment landscape of ROP by reducing the reliance on RPC and decreasing the extent of laser treatment. IVR provides a targeted, tissue-sparing therapeutic approach that preserves retinal structure and may reduce the risk of progression to advanced disease, including RD. These findings suggest that IVR represents an effective and less destructive treatment option for ROP and support its increasing role as a first-line therapy in appropriately selected patients. Further prospective studies are warranted to confirm its long-term safety and efficacy.
